# Erratum to “Nutritional and Lifestyle Interventions for Age-Related Macular Degeneration: A Review”

**DOI:** 10.1155/2017/2435963

**Published:** 2017-09-24

**Authors:** Ângela Carneiro, José Paulo Andrade

**Affiliations:** ^1^Department of Surgery and Physiology, Ophthalmology Unit, Faculty of Medicine of University of Porto, Porto, Portugal; ^2^Service of Ophthalmology, Hospital S. João, Al. Professor Hernâni Monteiro, 4200-319 Porto, Portugal; ^3^Department of Biomedicine, Anatomy Unit, Faculty of Medicine of University of Porto, Al. Professor Hernâni Monteiro, 4200-319 Porto, Portugal; ^4^Center for Health Technology and Services Research (CINTESIS), Faculty of Medicine, University of Porto, Rua Dr. Plácido da Costa, 4200-450 Porto, Portugal

In the article titled “Nutritional and Lifestyle Interventions for Age-Related Macular Degeneration: A Review” [1], there were a number of language errors, due to publisher error. The corrected version of the article is shown below:

## Abstract

Age-related macular degeneration (AMD) is the leading cause of blindness in the developed world. In this narrative review, we will summarize the nutritional interventions evaluated in numerous observational studies and a few randomized clinical trials. The AREDS and AREDS2 studies demonstrated that supplements including vitamins C and E, beta-carotene, and zinc may reduce the progression to advanced AMD in some patients by 25% over five years. This is one of the few nutritional supplements known to have a beneficial effect in any eye disease. Lutein/zeaxanthin supplementation may have beneficial effects in some individuals, whereas omega-3 fatty acids supplementation needs to be further investigated and supported by more evidence. Genetic factors may explain the different patterns of response and explain differences found among individuals. More importantly, a combination of lifestyle behaviors, such as the avoidance of smoking, physical activity, and the adoption of a healthy dietary pattern like the Mediterranean diet, was associated with a lower prevalence of AMD. The adoption of these lifestyles may reduce the prevalence of early-stage AMD and decrease the number of individuals who develop advanced AMD, consequently lowering the onerous and climbing costs associated with treatment of this disease.

## 1. Introduction

Age-related macular degeneration (AMD) is the leading cause of blindness in Western countries according to the World Health Organization [1] and this disease can significantly reduce quality of life [2]. As central vision is lost in advanced AMD, aging patients suffer limitations in their ability to function independently, because they have a diminished ability to recognize faces and to read the small print in newspapers, on food packages, and on medication labels [3].

The early stages of AMD are characterized by the presence of pigmentary abnormalities and drusen near the fovea [1]. Late-stage AMD has two forms: (a) geographic atrophy, with major loss of the retinal pigment epithelium (RPE) and choriocapillaris; (b) neovascular AMD, with newly formed blood vessels in the macular region that lead to leakage of blood and serum, causing irreversible damage and progressive vision loss [4].

The prevalence of AMD increases sharply with age, but despite major geographical and lifestyle differences it appears to be similar in European ancestry populations from the United States, Australia, and Europe [5, 6]. In a meta-analysis performed in 2004 by Friedman and collaborators, the prevalence rates for the late forms of the disease increase from less than 0.5% in subjects aged 50–60 years to 12–16% in individuals aged 80 years and above. Concerning early-stage AMD, an increase from about 1.5% in those with European ancestry aged 40–49 years to more than 25% in individuals aged 80 years and over was found [5].

In the recent past, AMD was an ignored and untreated disease of the elderly. Now, hundreds of millions of dollars from the healthcare budgets of Western countries are spent on new treatments for the neovascular form. According to a 2013 United Nations report concerning the aging of the world population, the percentage of people aged 60 years or older is growing faster than any other age group due to fertility declines and increasing longevity. With these longer lifespans, the prevalence of AMD is also rising in the rest of the world outside Western countries. In Asia, the number of people with AMD increased in the last two decades, doubling the number of cases reported globally [6, 7]. For example, in Japan the prevalence of neovascular AMD has changed drastically. AMD was not registered in 1994 and now is the fourth-leading cause of visual disability [8], although it remains relatively low compared with Western countries [8].

There are therapies for neovascular AMD, but no effective treatment exists for early-stage AMD and geographic atrophy, which is dramatic in terms of public health over the next two decades [9]. Therefore, there is considerable interest in preventing and delaying the onset of AMD by identifying and modifying risk factors [10]. Disease progression is slow and randomized multicenter clinical trials are extended in time, expensive, and hard to perform and analyze. However, even a modest protective effect on the prevention and/or progression of AMD would have a significant impact on patient welfare and on the burden to society.

## 2. Risk Factors

AMD is a complex disease with numerous inherited risk factors, including one major genetic risk locus on chromosome 1 in the complement factor H region and a second locus in the HTRA/ARMS2 region on chromosome 10, in parallel with minor genetic factors identified through genome-wide association studies [10–12].

Similar to genetic polymorphisms and a family history of AMD, increasing age is another very important, consistent, and non-modifiable trait. Aging is clearly associated with an exponential rise in the incidence and prevalence of AMD [13]. Other non-modifiable traits associated with an increased risk of AMD in the literature are light skin color, light iris color [14, 15], and perhaps being female [6, 16]. These risk factors guide the development of new therapies and novel treatment strategies, but the present situation demands efforts to identify and alter modifiable risk factors.

Modifiable risk factors were found using epidemiological studies. It is important to stress that single epidemiological studies cannot be interpreted when isolated from other evidence, unless they can be replicated in independent cohorts. Biological plausibility based on the pathophysiology of the disease added to the associations found in several epidemiological studies adds confidence in the results, but does not prove the existence of causality [10].

Epidemiological studies all around the world provided important information concerning the distribution of the different patterns of the disease in several countries and identified several environmental and modifiable risk factors. The most recognized environmental risk factor for AMD is smoking, which has a dose-dependent relationship. The risk of late-stage AMD is multiplied by 2.5 to 4.5 and appears to be cumulative over time [17, 18]. On the other hand, smoking and other environmental factors were not clearly associated with early-stage AMD [17, 19–21].

Curiously, as AMD shares several characteristics with Alzheimer's disease (AD), such as intra- and extracellular deposits, it was called the “Alzheimer's of the eye” [22, 23]. Additionally, AMD closely mirrors AD in terms of lifestyle and many risk factors (aging, hypertension, smoking, obesity, hypercholesterolemia, and arteriosclerosis). However, the similarity is relative, as the genetic background is completely different [22, 23].

As there are no effective means of primary prevention other than smoking cessation, other possible changes in lifestyle including nutritional changes acquire enormous importance. Multiple studies revealed oxidative stress as one of the mechanisms implicated in the pathogenesis of AMD and diet is generally the main source of antioxidants [24]. Concerning eye tissues, their biological integrity is dependent on the balance between the production of free radicals and their catabolism [24, 25].

## 3. The Human Eye and the Oxidative Stress

The production of free radicals increases with age, but some of the endogenous defense mechanisms decrease, creating an imbalance that leads to progressive damage of cellular structures [25]. The vast antioxidative network includes vitamins (C, D, and E), enzymes (superoxide dismutase, catalase, and glutathione peroxidase), carotenoids (alpha- and beta-carotene, lycopene, lutein, and zeaxanthin), and many other compounds (flavonoids, lipoic acid, uric acid, selenium, and coenzyme Q10) [26]. They act as a protective chain, and the different antioxidants have a synergistic effect and protect each other from direct destruction in the process of neutralizing free radicals [25]. The generation of singlet oxygen free radicals will extract hydrogen atoms from molecules with available six double bonds, such as the omega-3 fatty acid docosahexaenoic acid (DHA), leading to lipid peroxidation [24]. The lipid peroxides cross-link with proteins, nucleic acids, and other compounds, adversely affecting tissue structure and function [27].

The eye presents an antioxidant system, with multiple components intervening from the aqueous humor to the retina. The retina possesses an antioxidant system with several components, but vitamins C and E and the carotenoids lutein and zeaxanthin are the most important [28]. Both vitamins cooperate to decrease the retinal epoxide adducts and participate in protection from blue light-induced damage [29]. The carotenoids are concentrated in the plexiform area of the macula, but zeaxanthin predominates in the central fovea whereas lutein is mostly present in the periphery. Zeaxanthin is therefore a more effective antioxidant in the area where the risk of oxidative damage is higher [30]. In addition, both carotenoids are found in the outer segments of rods and probably of cones, which are particularly rich in DHA and therefore potentially vulnerable to lipid oxidation [28, 31].

DHA comprises 40% of the polyunsaturated fatty acids (PUFAs) in the brain and 60% of the lipid constituents of retinal photoreceptor membranes; in humans, DHA is obtained from the diet [28, 32]. Also, DHA may be converted from eicosapentaenoic acid (EPA), but this synthesis involves several steps including elongation and peroxisomal beta-oxidation [33]. DHA and EPA have pleiotropic effects through signal transduction, gene regulation, and plasma membrane dynamics [34]. Health benefits arise from the ability of DHA and EPA to reduce the production of inflammatory eicosanoids, cytokines, and reactive oxygen species [35] and modulation of the expression of numerous genes involved in inflammatory pathways [36]. DHA is present in small quantities in most tissues, but is a major structural lipid of the retina presenting in particularly high levels in this neural tissue [37, 38]. DHA may be involved in the permeability, thickness, fluidity, and other properties of the membrane of photoreceptors [38] and its insufficiency is linked to changes in the function of the retina [38, 39]. The anti-inflammatory actions may inhibit the formation of new choroidal vessels that appear in exudative AMD [39, 40]. EPA and other major dietary omega-3 fatty acids appear to have a similar action [38]. The renewal of retinal membranes demands a steady supply of these omega-3 fatty acids by RPE cells. If there is an imbalance in the retinal lipids, photoreceptor degradation and accumulation may lead to the formation of drusen, formed mainly by the debris of lipids and lipoproteins in the RPE layer and sub-RPE space [34].

## 4. Pathogenesis of AMD and Oxidative Stress

Although the exact pathogenesis of AMD is not known oxidative stress is considered to be involved, because the human eye, and particularly the retina, is exposed to the production of free radicals leading to a pro-oxidative environment [28]. This vulnerability is related to several factors: (a) high vascularization, which results in high oxygen tension; (b) light-induced stress in the fovea due to increased cellular density and metabolism; (c) retinal tissues that have a high quantity of unsaturated fatty acids and photosensitizing compounds, which are highly susceptible to oxidation; (d) the exposure of the retina to cumulative radiation [24, 28, 41].

The putative mechanisms are light-initiated oxidative damage and a reduction of the macular pigment [42, 43]. Long exposure to bright light, oxidative damage and the presence of oxidized metabolites in the outer segment of photoreceptors of the RPE may contribute to the formation of the drusen and pigment disturbances in the macula [44, 45].

It is therefore conceivable that dietary antioxidants and/or supplements may be beneficial to preventing and/or delaying the progression of AMD due to the decrease of oxidative stress and reduction of inflammatory events [28].

## 5. Diet and the Ocular Antioxidant System

In Western countries, the dietary intake of vitamins, carotenoids, essential fatty acids, and other oligoelements is generally enough to supply the needs of healthy individuals. The major dietary sources of the 18-carbon fatty acids alpha-linolenic acid (ALA) and linoleic acid (LA) are vegetable oils [26]. The ingestion of these fatty acids is essential as humans do not possess the enzyme systems necessary to insert double bonds at the omega-3 or omega-6 positions [26, 46]. Diets rich in fish, meat, and eggs will provide EPA, DHA, and arachidonic acid, but they can also be synthesized from ALA and LA [24, 47]. However, the conversion rate of ALA to omega-3 PUFAs is relatively low in humans and involves several steps [48]. Due to this, oils rich in ALA do not significantly increase DHA and EPA levels [33, 46, 48].

In contrast, lutein and zeaxanthin are not synthesized by humans. They are found in plants such as fruits and spices (lutein) and lettuce, broccoli, and spinach (zeaxanthin) [10].

The role of nutrition and nutritional supplements has been raised by a number of observational and randomized clinical trials. Three types of nutritional factors have mainly been investigated for their potential protection against age-related eye diseases: antioxidants (mainly vitamins C, E, and beta-carotene), zinc, the carotenoids lutein and zeaxanthin, and omega-3 PUFAs. The results of the most important randomized clinical trials concerning the effects of these nutritional factors on AMD are presented and discussed below [28].

## 6. The Age-Related Eye Disease Study (AREDS)

AREDS was a complex randomized clinical trial designed to evaluate the effect of vitamins C (500 mg), E (400 IU) and beta-carotene (15 mg), with or without zinc (80 mg), and copper (2 mg), on the progression of AMD [28, 49]. Most of the 4757 patients, aged 55 to 80 years, were taking nutritional supplements at the time of study enrollment and, to standardize this a daily dose of a multivitamin and minerals tablet (Centrum®) was provided and 66% of the participants opted to use this supplement. The supplements were provided in much higher concentrations than the recommended daily intake [50]. Therefore, the effects found were attributed to the AREDS supplements [50]. The patients were followed up for a mean period of 6.3 years [28].

The form of vitamin E used in AREDS was alpha-tocopherol, the form that is mainly present in tissues and blood [51]. Vitamin E exists as eight fat-soluble compounds of tocopherols and tocotrienols, and each subgroup has several subtypes (alpha, beta, gamma, and delta). Nuts and seeds, whole grains, and dark leafy vegetables (spinach, collard greens) are rich sources of alpha-tocopherol, which is absorbed and accumulated [50, 51]. The dose used in the AREDS trial was 400 UI daily, whereas the recommended daily intake is only 22.4 UI [50].

Beta-carotene is a carotenoid often referred to as provitamin A [51]. Due to its antioxidative properties, beta-carotene was included in the multivitamin supplements, but a solid role in the prevention of AMD was not strongly supported [51]. Also, due to its effective antioxidant activity, vitamin C (ascorbate) was included in the AREDS formulation. However, there was no association between vitamin C intake and AMD in the Pathologies Oculaires Liées à l'Age (POLA) study or in the Eye Disease Case-Control Study Group [51–53].

At the beginning of the study, 1117 patients had few if any drusen (Category 1) and 1063 patients had extensive small drusen, pigment abnormalities, or at least 1 intermediate size drusen and were classified as Category 2. Category 3 included 1621 participants possessing extensive intermediate drusen, geographic atrophy not involving the center of the macula, or at least one large drusen. Finally, 956 participants were included in Category 4 as they had advanced AMD or visual acuity less than 20/32 due to AMD in one eye [28]. The results showed that treatment with zinc alone or in combination with antioxidants reduced the risk of progression to advanced AMD in patients in Categories 3 and 4 [28]. The reduction of risk at five years for those taking the supplements plus zinc was 25% when compared to placebo controls. The treatment effect persisted following five additional years of follow-up after the end of the clinical trial [54]. During the study, 407 participants without geographic atrophy at baseline developed, at least, moderate geographic atrophy, not necessarily involving the center of the macula [28]. No significant differences among treatments with antioxidants, with zinc, or antioxidants plus zinc on the progression of AMD were found [49].

After the publication of the AREDS report in 2001, the AREDS formulation became the standard of care. Due to serious concerns about the safety of beta-carotene, which increased the risk of lung cancer in smokers, this compound was replaced in the randomized clinical trial known as AREDS2 [28]. In addition to this concern, it was found that beta-carotene was not detectable in the human retina contrary to lutein, zeaxanthin, and mesozeaxanthin [10]. Moreover, in the Beta-Carotene and Retinol Efficacy Trial (CARET) study, the combination of retinol (vitamin A) and beta-carotene increased the risk of lung cancer and cardiovascular events [55]. This evidence discourages the use of beta-carotene in the prevention of AMD due not only to the side effects, but also to poor efficacy [51]. In addition, vitamin A alone was found to have no solid results concerning the prevalence of AMD [51] and it was not included in the original AREDS formulation.

## 7. The Age-Related Eye Disease Study 2 (AREDS2)

AREDS2 was a phase 3 study and controlled clinical trial, involving 4203 patients aged 50 to 85 years [56]. Conducted in 2006–2012, the selected participants were at risk of progression to advanced AMD with bilateral large drusen or large drusen in one eye and advanced AMD in the other eye [28, 56]. The main aim of AREDS2 was to improve the original AREDS formulation, making it more effective and safer. The lutein/zeaxanthin daily dose chosen was in the ratio 5 : 1 (10 mg of lutein : 2 mg of zeaxanthin) based on a small-scale study [57, 58]. These daily doses are substantially higher than a typical Western diet (1-2 mg of lutein : 0.2 mg of zeaxanthin). Primary randomization considered four groups of participants: (a) lutein (10 mg) + zeaxanthin (2 mg); (b) fish oil (350 mg of DHA + 650 mg of EPA); (c) lutein + zeaxanthin + DHA + EPA; and (d) placebo [56].

The expected 25% incremental improvement over the original AREDS results was not obtained [56]. The addition of lutein + zeaxanthin, DHA + EPA, or both to the AREDS formulation did not further reduce the risk of progression to advanced AMD. In the analyses evaluating the patients assigned to lutein/zeaxanthin (2123) versus the patients not assigned to lutein/zeaxanthin (2080), an added 10% reduction was found in the risk of progression to advanced AMD in patients assigned to lutein/zeaxanthin versus those not assigned to lutein/zeaxanthin. However, the patients who received DHA + EPA did not show this effect. Lutein/zeaxanthin was not related to an increase in lung cancer incidence, contrary to beta-carotene that was associated with a rise in lung cancer in former smokers [28, 56]. Mesozeaxanthin is now available in the market, but there is no randomized clinical trial supporting its superiority to lutein/zeaxanthin [10].

In brief, the evidence on beneficial and adverse effects from AREDS2 and other studies suggests that lutein (2 mg) + zeaxanthin (15 mg) could be better than beta-carotene (15 mg) in AREDS-type supplements, particularly for former or current smokers. Other studies also found a correlation between high plasma levels of lutein/zeaxanthin and a low risk of AMD, such as the POLA Study and others [52, 59, 60].

Large-scale epidemiologic observational studies, such as the Eye Disease Case Control Study (USA), agree with the findings of beneficial effects of dietary intake of carotenoids [61] and several other studies demonstrated an increase in macular pigment associated with the intake of carotenoids [60, 62, 63]. There are other epidemiological studies with other conclusions. For instance, the Rotterdam study showed a reduced risk for AMD in subjects with high dietary intakes of beta-carotene, vitamins C and E, and zinc [64], while the Physicians' Health Study did not reveal benefits for vitamins C or E [65].

Interestingly, data from the United States differs from European data, which could be explained by differences in nutritional patterns or supplement intake. For example, the majority of the participants in the AREDS study used vitamin supplements (Centrum) in addition to the supplementation tested in the study [4]. The mean baseline plasma vitamin C was 62 *µ*M/L in AREDS participants, whereas it was 31.6 *µ*M/L in men and 40.5 *µ*M/L in women enrolled in the French POLA study [4, 66]. The plasma levels of vitamin C of this French study were comparable to those of the EUREYE Study implemented in seven European countries [67].

A systematic review of prospective studies of dietary intake found no evidence that diets high in antioxidant vitamins prevent AMD [68, 69]. More specifically, there is no evidence from randomized clinical trials that healthy people should take vitamin and antioxidant supplements to delay or prevent the onset of AMD [68]. Recently, it was reported that high dietary intake of folate was associated with a decreased risk of progression of geographic atrophy, using a large prospective cohort with a high number of incident cases [70]. This association could be modified by genetic susceptibility, particularly associated with C3 alleles [70]. In this same study, thiamin, riboflavin, niacin, and vitamins B6 and B12 were not significantly related to progression [70]. Folate is provided by green vegetables, fruits, nuts, beans, and peas, the same sources as lutein and zeaxanthin [70].

## 8. Zinc Supplementation

Zinc levels are high in ocular tissues, but the distribution is not uniform [47]. It is the most abundant trace metal in the retina and is preferentially located in the inner nuclear and PR layers, that is, the regions affected by AMD [71]. Total zinc concentration in the RPE is high enough to release reactive zinc in the high micromolar range [71]. In photoreceptors, the loosely bound zinc ions may play a role in regeneration of rhodopsin and in the phototransduction cascade [71]. The US Food and Drug Administration (FDA) recommends 11 mg/day of zinc for men and 8 mg/day for women. A daily replenishment of approximately 1% of total body zinc is obtained from the diet and prolonged periods of zinc depletion cannot be compensated [71]. Oysters and other seafood contain more zinc per serving than any other food [50]. Beef, poultry, and pork generally provide the necessary intake [50]. Although zinc is present in beans, cereals, and nuts, plant-based phytates may inhibit their absorption [50]. Therefore, if meat and seafood are scarce in the diet, the consumption of zinc may not be sufficient as the body does not store zinc [50, 71].

Zinc is a cofactor of many active ocular enzymes, including superoxide dismutase and catalase [8, 72]. Zinc also binds complement factor H, inducing large multimeric forms that lose complement 3b inhibitory activity [73]. This action can help to suppress the chronic inflammatory events in the retina that can lead to AMD [73]. In vitro studies suggested that zinc supplementation attenuates endothelial cell activation and may affect the progression of AMD [74]. In an animal model of light-induced retinal degeneration, zinc supplementation induced changes in gene expression, enhancing antioxidative retinal capacity and the reduction of oxidative damage [75].

In AMD, the content of zinc in human RPE/choroid is decreased by 24%. On the other hand, high levels of zinc are present in drusen, probably affecting the oligomineralisation of complement factor H [47, 71]. Also, the expression of some transport, sensory, and trafficking/storage proteins was found to be downregulated in AMD maculas [47, 71]. These changes in retinal zinc homeostasis justify its presence in the AREDS formulation and in other trials. As high doses of zinc may interfere with copper absorption, the AREDS formulation included a copper supplement (2 mg/day) [4].

A systematic review of these trials with zinc was published in 2013 [76]. Ten studies analyzing the effect of zinc intake, both from supplements and/or foods, in treatment and primary prevention were included: four randomized clinical trials, four prospective cohorts, and two retrospective cohort studies. The review found that current evidence on zinc intake for the prevention of AMD is inconclusive. However, AREDS revealed that 80 mg of zinc oxide, alone or in combination with antioxidants, significantly reduced the risk of progression to advanced AMD in individuals with moderate AMD. The risk of visual acuity loss was of a similar magnitude, but not statistically significant. The other two randomized clinical trials showed a statistically significant increase in visual acuity in early-stage AMD patients and the other one revealed no effect of zinc treatment on visual acuity in advanced AMD patients [76]. Furthermore, results from the remaining six cohort studies on associations between zinc intake and incidence of AMD were inconsistent. The authors of the systematic review concluded, based on the strength of AREDS, that zinc treatment may be effective in preventing progression to advanced AMD, but zinc supplementation alone may not be sufficient to produce clinically meaningful changes in visual acuity [76].

Due to possible secondary effects of high doses of zinc in the stomach, AREDS2 evaluated the effects of a lower dose of zinc (25 mg versus 80 mg), but no significant difference in AMD progression was reported [56]. Note that, as stated previously, most of the participants in both AREDS trials simultaneously took Centrum that included the recommended daily intake of zinc, increasing the daily dose of zinc [4]. As a trend favoring the higher dose of zinc was found [77], the present AREDS2 formulation includes 80 mg of zinc. It should be noted that the doses of daily zinc are much higher than recommended by the FDA, but the secondary effects described were minimal.

More recently, it was reported that response to AREDS supplements is affected by genetic factors and the effectiveness of antioxidant/zinc supplementation is influenced by genotype [78]. Particularly, in AREDS patients with the high-risk CFH/low-risk ARMS2 genotype, zinc-containing treatments may worsen outcomes for patients who have moderate to severe AMD [77].

## 9. Lutein and Zeaxanthin Supplementation

As already mentioned, the carotenoids, lutein/zeaxanthin, are concentrated at the central fovea composing the macular pigment [2, 42]. By scavenging reactive oxygen species and filtering potentially damaging blue light, they protect the macular region from photooxidative injury and can decrease a toxic byproduct of the visual cycle (A2E), stimulated by this blue light [10, 42]. Lutein/zeaxanthin supplements may offer protection to decrease the number of lipofuscin granules and increase the stability of lysosomes [42]. Using animal models, lutein/zeaxanthin preserved macular health and improved functional abnormalities [14]. The absorption of poorly focused short wavelengths by the macular pigment decreases chromatic aberration and improves visual resolution [15, 79].

Lutein/zeaxanthin supplementation from foods can increase pigment macular density, but this capacity varies among individuals [79]. Physical activity may directly contribute to a denser macular pigment due to the reduction of inflammation and oxidative stress, or indirectly by reducing obesity. In fact, obesity is related to a lower density of a macular pigment in this study and others and may increase oxidative stress [13]. The status of the macular pigment may be improved by a healthy diet and physical exercise [10, 79].

Despite strong biological plausibility, the epidemiological studies have not found consistent results [80]. More importantly, as stated above, the AREDS2 trial failed to confidently demonstrate protective effects of lutein/zeaxanthin in the primary analyses [56]. However, there was a 26% risk reduction when the analysis was restricted to a subgroup of participants at the bottom 20% of the dietary intake of lutein/zeaxanthin [56, 81]. This secondary analysis supports the hypothesis that supplements are effective only when the background dietary intake is below a sufficient threshold [81].

This hypothesis is supported by a long-running (two decades of follow-up), very large prospective cohort from the Nurses' Health Study and Health Professionals Follow-up Study that found an association between a higher intake of lutein/zeaxanthin and a 40% lower risk of advanced AMD [80]. The intake of carotenoids was not associated with intermediate AMD. This finding was interpreted as an effect on the progression of AMD but not on the initiation of the disease [80]. Although there is no causal inference, this is probably the best available evidence in the absence in the near future of a well-designed large-scale randomized trial like AREDS2 [80].

Some other studies found an association between carotenoids and intermediate AMD. However, only one of three prospective cohort studies reported a significant inverse association between the intake of lutein/zeaxanthin and intermediate AMD [42]. In late-stage AMD the dysfunction or loss of macular photoreceptors may not be rehabilitated [82]. A meta-analysis of eight high-quality randomized clinical trials involving 1176 AMD patients in which a comparison of lutein/zeaxanthin intervention with placebo was performed [42], with the outcomes of visual acuity, contrast sensitivity, glare recovery time, and subjective perception of visual quality, found that lutein/zeaxanthin improved visual acuity and contrast sensitivity of AMD patients and, more importantly, there was a dose-response relationship [42].

The intake of xanthophyll carotenoids is far below the recommended level and there is a tendency for a decrease in Western countries [83]. A recommendation for an increased intake of lutein/zeaxanthin from food sources or supplements should be advised, especially for AMD patients. The results of the Taurine, Omega-3 Fatty Acids, Zinc, Antioxidant, Lutein (TOZAL) study showed that AMD patients require at least six months of supplementation to have a positive outcome, such as changes in visual acuity and other visual parameters [84].

## 10. Omega-3 Supplementation

In AREDS2, the addition of DHA + EPA to the original AREDS formulation (vitamin C, vitamin E, beta-carotene, zinc, and copper) did not further reduce the risk of AMD. This finding was unexpected as epidemiological studies have for more than 10 years pointed to a beneficial effect of dietary omega-3 in the prophylaxis of AMD [85]. There is consistent evidence that the intake of high doses of DHA present in oily fish is associated with a decreased risk of neovascular AMD [32, 39]. Concerning the dietary consumption of n-3 fatty acids, the Eye Disease Case Control Study in the United States demonstrated an association between a higher intake of n-3 fatty acids and a lower risk of AMD among individuals on a diet low in LA [8, 25]. The Blue Mountains Eye Study in Australia demonstrated a protective effect of n-3 fatty acids in late-stage AMD in individuals in the highest quintile of intake [43]. Subjects in the AREDS trial who reported the highest consumption of n-3 fatty acids were also less likely to have neovascular AMD at baseline [44]. Other studies have reported an inverse association between the dietary consumption of n-3 fatty acids and neovascular AMD [44, 45, 57]. Some researchers criticize AREDS2, stating that the design, setting, and selection of patients in the AREDS2 trial did not allow for ascertaining the potential of omega-3 supplements [85], due to an inadequate duration of treatment, inadequate dose, or both [56, 85]. Another possible explanation is that the effect may be modified by underlying genetic risk factors [86, 87]. It is also possible that there is an optimal level of supplementation with omega-3 fatty acids and if the level is not optimal it could be ineffective or detrimental. A more appropriate conclusion is that in a well-nourished population AREDS2 was not more effective than the original formulation [22].

A prospective randomized prospective study, the Nutritional AMD Treatment-2 (NAT-2) trial, had a true placebo group and demonstrated that patients who achieved high red blood cell membrane EPA/DHA levels were significantly protected against AMD versus those patients with low levels of EPA/DHA [32, 39]. However, in the same NAT-2 study, with supplementation with 840 mg/day DHA (87 individuals) or placebo (80 individuals) for three years, dynamic drusen remodeling was not affected by DHA supplementation [34]. This drusen remodeling showed a tendency to be influenced by CFH genotype [34].

In an open-label experiment, it was reported that high doses of EPA (3.4 g) and DHA (1.6 g) on a daily basis for six months improved the visual acuity of patients with dry AMD [88]. Also regarding the intake of omega-3 PUFAs, a Cochrane meta-analysis published in 2012 and updated in 2015 concluded that there is currently no evidence to support the idea that increasing levels of omega-3 PUFAs in the diet prevents or slows the progression of AMD, in accordance with the AREDS2 trial [89, 90].

In short, the majority of evidence suggesting a positive effect of dietary omega-3 intake on the development and progression of AMD comes from observational studies and this remains to be demonstrated in randomized clinical trials [39]. In a recent paper, the systemic confounders that may affect the serum measurements of omega-3 and omega-6 PUFAs in patients with retinal disease were discussed [91]. In that pilot study, they demonstrate that serum lipid profiles are significantly altered by variables like fasting and medication. Patient fasting status may affect the serum levels of unsaturated and saturated fatty acid levels, influencing the results and conclusions of the studies [91].

Summing up the current evidence, it is reasonable to advise AMD patients to consume dietary fatty fish (e.g., salmon, tuna, sardine, mackerel, and trout) or fish oil supplements. It is prudent to inform the patients of the doubts concerning their efficacy and the lack of support from large randomized clinical trials.

## 11. The Mediterranean Diet

Dietary patterns where nutrients in the food can interact synergistically is a recent paradigmatic shift in nutritional sciences. Numerous studies show that a healthy diet, the maintenance of an adequate body weight, and an active lifestyle are important to maintaining health and avoiding the physical and cognitive degeneration associated with aging [3, 26, 92].

The Mediterranean diet is one of the most studied healthy dietary patterns. This diet is rich in vegetables and fruits, thereby providing high amounts of bioactive antioxidant compounds. Furthermore, olive oil and fatty fish rich in omega-3 fatty acids are present in the Mediterranean dietary pattern [26, 93].

From the scientific point of view, there are major differences between the Mediterranean diet and the so-called Western diet. In fact, the INTERHEART and INTERSTROKE studies [94, 95] considered the existence of three main dietary patterns, evaluated by a dietary risk score: (a) Western diet with high intake of fried foods, salty snacks, eggs, and red meat; (b) oriental diet, high in tofu and sauces like soy and others; (c) prudent diet with a high intake of fruits and vegetables with some characteristics of the Mediterranean diet. It was found that the Western diet increased the population risk for acute myocardial infarction and stroke by approximately 30%. In contrast, the prudent diet decreased the same risk by 30%. The oriental diet was neutral, probably because the high intake of fruits, fish, and vegetables was offset by the high salt intake and other factors [26, 94, 95].

Defining a Mediterranean diet is difficult considering that the geographical region includes more than 17 countries [26]. According to De Lorgeril, the Mediterranean dietary pattern is inspired by the traditional diet found in Greece and Southern Italy [26]. It has the following characteristics: (a) high consumption of fruits, legumes, and other vegetables, bread and other cereals, potatoes, beans, nuts, and seeds; (b) olive oil is the main source of monounsaturated fat; (c) dairy products, fish, and poultry are consumed in low to moderate amounts; only low quantities of red and processed meat are present; (d) wine is consumed during meals in low to moderate amounts [26, 96].

Summarizing the prospective observational studies, a meta-analysis was published by Sofi and collaborators in 2008 and updated in 2010 [97, 98]. The studies prospectively analyzed the association between adherence to a Mediterranean diet, mortality, and incidence of diseases, with a total of more than 2 million subjects followed from 3 to 18 years. Greater adherence to a Mediterranean diet was associated with an improvement in health status, namely, a major reduction in overall mortality (9%), mortality from cardiovascular diseases (CVD) (9%), and incidence of mortality from cancer (6%). The reduced incidence of Parkinson's and Alzheimer's diseases (13%) demonstrated that the adherence to the Mediterranean diet prevented these major chronic neurodegenerative diseases associated with aging [26, 97, 98].

The* Prevención con Dieta Mediterránea* (PREDIMED) trial, a multicenter randomized clinical trial in Spain [99], showed that relatively small changes in diet have beneficial effects [99]. The clinical trial included 7447 subjects at risk of CVD. The conclusions demonstrated that the adoption of a Mediterranean-style diet reduced the risk of cardiovascular complications by 30%, including a reduction of the risk of stroke by 40% over a follow-up of approximately five years [99]. In 2013, the Cochrane Heart Group published a review analyzing the Mediterranean dietary pattern for the primary prevention of CVD [100], concluding that the Mediterranean diet may reduce some cardiovascular risk factors (total cholesterol levels, LDL cholesterol levels) [100]. Additional randomized clinical trials are necessary, but there is sufficient scientific evidence to recommend the Mediterranean dietary pattern to individuals who want to age in good health while still appreciating food [26, 101].

## 12. Mediterranean Diet and AMD

Assessing dietary patterns with respect to AMD is relatively recent. Association of a particular nutrient to AMD is difficult to dissociate from other aspects of the diet. More importantly, there are synergistic relationships between the food components [3, 26, 86].

In the Carotenoids Age-Related Eye Disease Study (CAREDS), high adherence to a Mediterranean diet was associated with a lower prevalence of early AMD [86]. Previously, it was reported that advanced AMD was related to overall diet quality using the Health Eating Index (HEI) [102]. Data from the Melbourne Collaborative Study showed that a diet rich in fruits, vegetables, nuts, and chicken was associated with a lower prevalence of AMD [103]. Conversely, the Western diet was associated with increased odds of AMD [104]. The oriental pattern of diet was linked to decreased odds of AMD [104].

Merle and collaborators used the data from 2525 individuals from the AREDS trial and dietary information collected from food-frequency questionnaires and the alternate Mediterranean diet score [86]. This score was constructed from individual intakes of vegetables, fruit, legumes, whole grain, nuts, fish, red and processed meats, alcohol, and the ratio of monounsaturated to saturated fats. They reported that a high score was associated with a 26% reduced risk of progression to advanced AMD after adjustments for demographic, behavioral, ocular, and genetic factors. The consumption of fish and vegetables was associated with a lower risk of progression to advanced AMD [86]. The administration of AREDS supplements did not change the protective effect of the Mediterranean diet on the risk of progression [86]. In addition, they reported that the Mediterranean score was associated with a lower risk of advanced AMD among individuals carrying the CFH Y402H non-risk (T) allele. CFH is one of the main genes implicated in AMD and the risk allele leads to complement activation, increasing AMD risk. The authors hypothesize that the effect of the Mediterranean diet is stronger among subjects with the non-risk allele [86]. The biological plausibility of the benefits of the Mediterranean diet involves a decrease in oxidative stress and inflammation. Accordingly, individuals with higher adherence to the Mediterranean diet have higher plasma concentrations of some important and beneficial biomarkers [105].

In a population in the central region of Portugal, it was reported that higher adherence to a Mediterranean diet score was associated with a lower AMD risk [6]. In this Coimbra study, the consumption of fruit, beta-carotene, and vitamin E was particularly beneficial [6]. In an update of the study presented at the American Academy of Ophthalmology meeting (Chicago, 2016), it was reported for the first time that coffee, also rich in antioxidants, was protective. Of the participants in this study who consumed caffeine (one cup of espresso, approximately 78 mg of caffeine), 54.4% did not have AMD (data not published).

As one of the key components of the Mediterranean diet, olive oil was studied in the ALIENOR (Antioxydants, LIpides Essentiels, Nutrition et maladies OculaiRes) study, a population-based study aimed at verifying the associations of nutritional factors such as antioxidants, macular pigment, and fatty acids [106]. After adjustments for the multiple potential confounders, there was a decreased risk of late-stage AMD among olive oil users in 654 subjects (1269 eyes) with a mean age of 72.7 years, with complete data suggesting a protective role of olive oil consumption for late-stage AMD in an elderly French population [106]. On the other hand, no association was found between olive oil consumption and early-stage AMD, and the use of other types of fats was not associated with any stage of AMD. Similar results were found in an Australian cohort (6734 individuals aged 63.7 years on average at baseline), where an inverse association between the intake of olive oil and the prevalence of late-stage AMD was found [107].

Olive oil is a mixture of lipids and antioxidant compounds and it contains monounsaturated fatty acids (MUFAs) and polyphenols that have strong antioxidant and anti-inflammatory properties [86]. A laboratory study in ARPE-19 human retinal pigment epithelial cells reported that the major polyphenol of olive oil, hydroxytyrosol, prevented the degeneration of retinal pigment epithelial cells induced by oxidative stress [108]. The protection probably does not rely on oleic acid, the main MUFA component, as the associations between MUFA intake and AMD are inconsistent in the literature. It is suggested that the protection derives from phenolic compounds, including oleocanthal, hydroxytyrosol, and oleuropein [106]. However, more studies are needed to find the mechanism by which olive oil has beneficial effects on late-stage AMD [106].

No association was found between oils rich in omega-3 and any stage of AMD. The ALIENOR study also found no association between olive oil consumption and early-stage AMD [106]. This lack of association suggests that the risk factors are dissimilar at different stages of the disease. Some risk factors may favor the accumulation of drusen and the pigmentary disturbances. Others may have an influence on neoangiogenesis and/or apoptosis. Another explanation, as suggested by some studies, is that early-stage AMD may represent a very heterogeneous group of patients, and some of these patients present a low risk of developing late-stage AMD [109–111].

## 13. The Genetic Risk, Lifestyle, and AMD

In epidemiological studies a healthy diet, avoiding food high in sugar, fat, alcohol, refined starch, and oils, the absence of smoking, and physical activity (low-intensity exercise for one or two hours per day, outside when possible) were associated with a reduced occurrence of early-stage or advanced AMD, or both [3]. This risk reduction was greater when multiple lifestyles were considered together, as shown by CAREDS [3]. In this study, women aged 50–74 years who displayed a combination of healthy lifestyle factors such as healthy diet, physical exercise, and not smoking had threefold lower odds for early-stage AMD when compared to women who displayed unhealthy lifestyles. The several mechanisms of the healthy lifestyle that lead to protection are difficult to disentangle from one another [3]. The energy expenditure of the physical exercise leads to an increase in daily nutrient intake. Both physical activity and diet may contribute to better vitamin D status, also associated with lower risk of AMD [112].

Insufficient physical activity increases the occurrence of numerous metabolic and vascular diseases [113] and may facilitate the progression of some cases of AMD [113, 114]. Sedentarism may contribute to increasing inflammatory events and dysfunction of the endothelium [113]. This hypothesis is supported by the finding that elevated C-reactive protein in patients with neovascular AMD is partly explained by physical inactivity [115]. On the contrary, physical activity can upregulate the enzymatic systems related to antioxidant protection, reducing oxidative events [113].

There is another important factor that can modify the benefits of the healthy lifestyles: genetic risk [116]. In fact, in the Unites States, the Y402H (rs1061170) variant within the complement factor H (CFH) gene and the A69S (rs10490924) variant within the age-related maculopathy susceptibility 2 (ARMS2) locus increased the risk (1.5- to 3-fold) for both early-stage and late-stage AMD in individuals of European ancestry [116, 117]. Wang and collaborators reported that the ingestion of lutein only reduces the incidence of AMD among persons with two or more alleles from common CFH and ARMS2 variants [118]. Meyers and collaborators studied the joint associations of diet, lifestyle, and genes in AMD and concluded that physicians should recommend the adoption of healthy lifestyles at early ages in individuals who have a family history of AMD and also they should motivate AMD patients to follow the same advice [116].

Stressing that the benefits and safety of the use of high-dose antioxidants for long periods in the prevention or delay of the progression of AMD in early stages has not been established [119], the adoption of healthy lifestyles at early ages may ultimately significantly benefit the patients and families at genetic risk. Public health interventions to consume plant-rich, high lutein diets, physical activity, and cessation of smoking are recommended strategies for AMD prevention. These recommendations are particularly important in individuals at genetic risk, with a family history of AMD or both. The combination of healthy lifestyles practices may be more important in the reduction of AMD than a focus on one. Collectively, these changes may reduce oxidative stress, blood pressure, and blood lipids. In short, they may reduce the systemic inflammation that contributes to the pathogenesis of AMD and other chronic diseases, including neurodegenerative diseases, although there is a lack of clinical trials to give solid evidence for these benefits [116]. These recommendations can lead to an improvement of outcomes through genotype-directed therapy [12]. The knowledge of modifiable risk factors along with information concerning genetic risk variants for AMD has greatly improved the management of patients and the ability to predict which patients will develop or progress to advanced forms of AMD [87]. Individualized prevention and treatment strategies and personalized medicine are becoming a reality [87].

Changes in lifestyle, such as eating a healthy diet like the Mediterranean diet, and if necessary complementing dietary patterns of selected individuals with appropriate supplements, may play an important role and avoid progression to advanced stages of AMD [86]. There are good rates of compliance with dietary advice; that is, patients change their diet, but patient education by health professionals can be improved [120].

## 14. Conclusion: Nutritional Advice to Patients with AMD

The nutritional advice of clinicians to their patients must be balanced and they should discuss what is supported by scientific evidence and the current doubts. This advice should be supported by high levels of evidence, preferably from randomized clinical trials [119].

Current evidence shows that all AMD patients should be advised to increase their consumption of green leafy vegetables and to eat oily fish, at least twice a week. A Mediterranean type of diet may provide additional benefits in other age-related diseases beyond AMD [26].

Patients with moderate or advanced AMD should be advised to use AREDS-based supplements. Current smokers or ex-smokers are advised to avoid formulations with beta-carotene [120].

If patients are unsure of their dietary lutein intake, they should be advised to use lutein/zeaxanthin AREDS-based supplements, given the lack of side effects found in most trials [120]. If they have a normal or high dietary lutein intake they can consume the modified beta-carotene free AREDS formulation [120].

Dietary modifications may not only delay the onset of AMD, but can also slow the progression of the disease. Supplementation with AREDS-based supplement slows the progression of AMD, but does not prevent its development.

More importantly, the adoption of a Mediterranean diet and physical activity and avoiding smoking and sedentary behavior may reduce the prevalence of the early stages of AMD, decrease the number of individuals who develop advanced AMD, and consequently reduce the burden associated with the treatment of this disease.
